# Development of microparticles for oral administration of *Periplaneta americana* extract to treat ulcerative colitis

**DOI:** 10.1080/10717544.2022.2112115

**Published:** 2022-08-18

**Authors:** Meng Li, Hao Wu, Shuang Wang, Shengshun Wu, Jing Han, Yang Han

**Affiliations:** a School of Pharmaceutical Engineering, Shenyang Pharmaceutical University, Liao Ning, China; b School of Functional Food and Wine, Shenyang Pharmaceutical University, Liao Ning, China; c School of Traditional Chinese Medicine, Shenyang Pharmaceutical University, Liao Ning, China

**Keywords:** *Periplaneta americana*, ulcerative colitis, oral drug administration, Eudragit S100, microparticles, polypeptide

## Abstract

Ulcerative colitis (UC) is a chronic disease, which can result the inflammation of the rectum, mucosa of the colon, and submucosa. The active component such as polypeptide in *Periplaneta americana*, which is one of the most common insects in the nature, can be extracted to treat UC. However, the active components in *Periplaneta americana* extract (PAE) can be degraded in the stomach due to its extreme acidic environment and enzyme. In this study, we developed a pH-dependent drug delivery method using polymer cellulose acetate (Eudragit S100) as a carrier to deliver high concentration PAE to inflamed colon. Both in vitro and in vivo results showed the PAE-Eudragit-S100 could treat UC through delivering active drug components to colon without degradation.

## Graphical Abstract

Eudragit S100 polymer solution was mixed with PAE ethanol solution by sonicating. Then mixture was then mixed with lecithin and glycerol monooleate in a blender . The emulsion was stirred at room temperature with a magnetic stir bar at 420 rpm. Them the microparticles were freeze-dried in a freeze-dryer. Finally, When mice were treated with this drugs. the drugs were not decomposed in stomach and small intestine but damaged in the colon, which had a good curative effeet on colitis.

**Figure:**
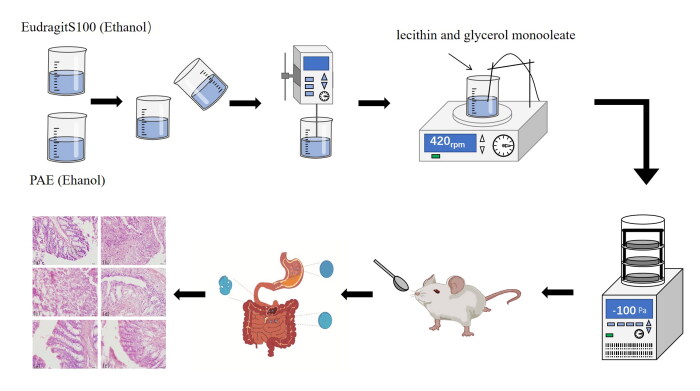

## 1. Introduction

UC is a chronic disease, which can cause the inflammation of the rectum, mucosa of the colon, and submucosa (Rubin et al., 2019; Ungaro et al., 2019; Sarvestani et al., 2021). Patients who have UC are always suffered abdominal pain, diarrhea and bloody stools. The incidence of UC is related to genes mutation such as NFKBIZ, ZC_3_H_12_A, PIGIR (Nanki et al., 2020) and MDR1 (Dragana et al., 2018), infection of bacteria (Kushkevych et al., 2017; IvanKristýna et al., 2021), stress and socio-economic factors and other factors (Carbonnel et al., 2009; Cortot et al., 2009). In addition, UC also increases the risk of patients having cancer (Jonsson et al., 2010; Den et al., 2019). To treat UC, it requires patients to take drug for a long period of time which are always related to drug adverse response and poor compliance of patients. The main adverse effects that are reported after using conventional therapy of UC include fever, nausea, headache, kidney damage, myopathy, myalgia, edema, neoplasia, congestive heart failure, tuberculosis, tremor, and hirsutism (Yoko et al., 2014). Bronchitis, arthralgia, headache, dizziness, abdominal cramps, and minor metabolic disorders would happen when the patients were treated with 5-aminosalicylates (Patil & Moss, 2008; Miehlke et al., 2014). Corticosteroids, though effective for UC when immediate remission is required, also induce some side effects, including edema, moon face, acne, mood disturbances, adrenal suppression, congenital fetal abnormalities, cushingoid face, gastric ulceration, and osteoporosis. Moreover, the routine use of corticosteroids may cause cataract and hyperglycemia, and a huge surge in the chances of getting severe relapse (Hanauer, 2008; Tiago et al., 2012; Kondamudi et al., 2013).

The *Periplaneta americana* is the one of the most common cockroaches in US. Although the wild cockroach become a public health problem due to its ability to spread virus, fungi and bacteria to human being, the body of cockroach is rich of many kinds of nutrients such as protein, amino acids and nucleotides (Lee et al., 2011; Yun et al., 2017; Dingchun et al., 2018). The dry cockroach body is recorded as a traditional Chinese medicine in ‘Shennong Materia Medica Classic’, which is commonly used for infantile malnutrition, sore throat, insect and snake bite, sore carbuncle, ulcer of digestive tract, etc (Chen et al., 2019). Studies have shown that the *Periplaneta americana* extract (PAE) promotes the growth of new granulation tissue and repairing ulcer wounds (Jing et al., 2019; Li et al., 2019), which can be used to treat gastric ulcers (Ma et al., 2018; Lu et al., 2019). PAE can be delivered to treat UC intravenously or subcutaneously in mice by increasing epidermal growth factor, which significantly reduces the lesion area (Xue et al., 2020). However, subcutaneous and intravenous administration of drugs always induces severe infusion-related reactions and adverse reactions, which significantly limits the clinical practice of PAE (Soeters & Aus, 1989). To lower the risk of subcutaneous or intravenous administration without scarifying drug efficacy, oral administration of PAE is an alternative delivery approach. But most of the active ingredients, such as polypeptide and protein molecules, may be degraded in the stomach before entering the colon (Abuhelwa et al., 2017; Cao et al., 2019). To deliver PAE to the inflamed colon without being degraded in gastric environment, an advanced drug delivery system should control the release of PAE in different environments (Li et al., 2020).

**Figure 1.**
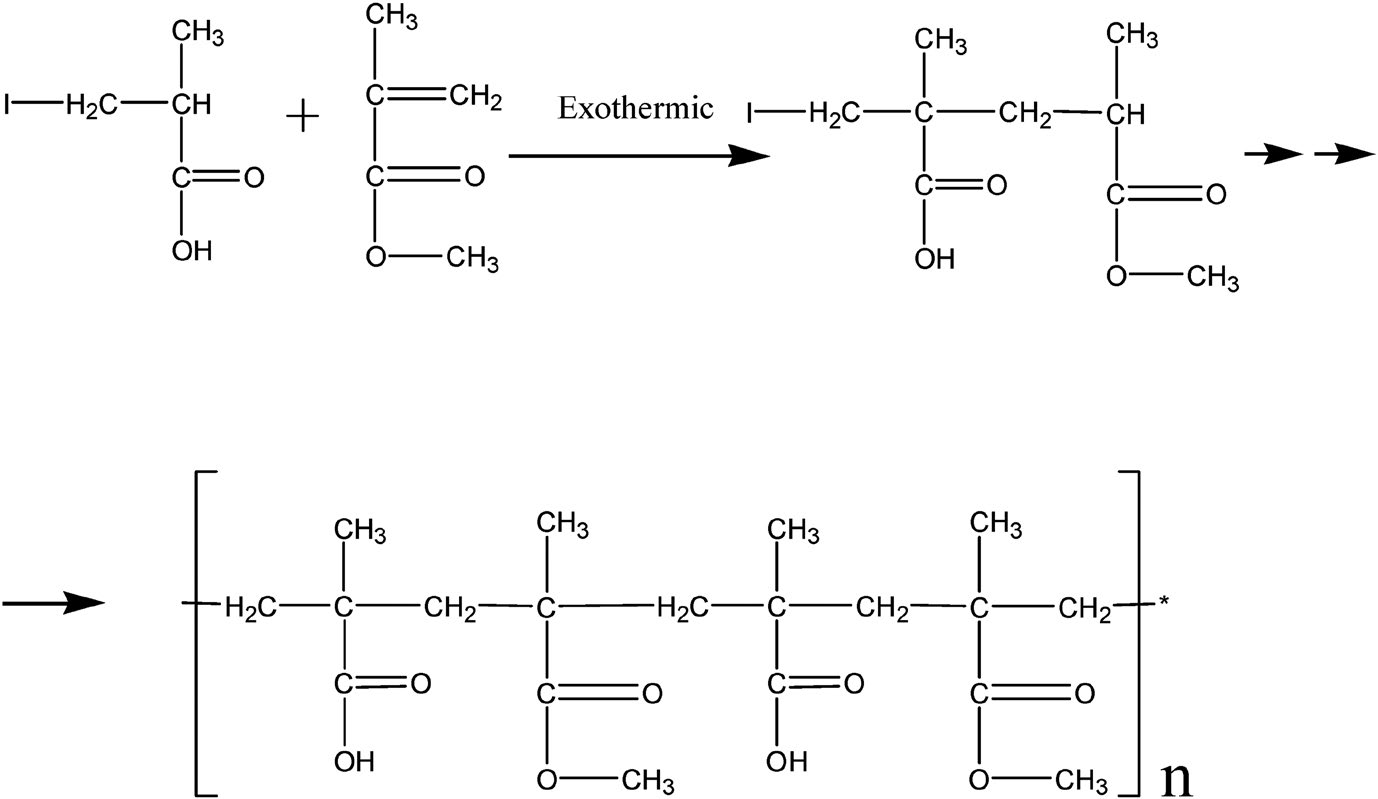
Chemical structure of Eudragit S100.

**Figure 2.**
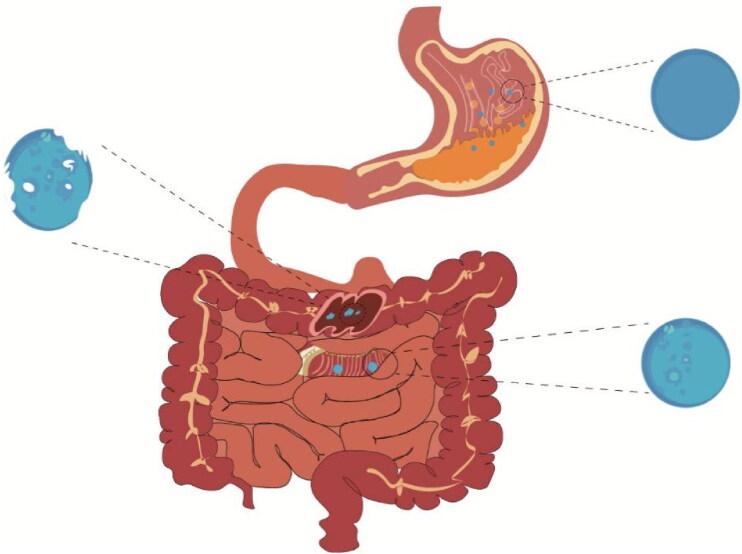
Release of microparticles in body.

In this study, we developed a pH-dependent drug delivery method using Eudragit S100 (chemical structure is shown in Figure 1) as a carrier to deliver the PAE to the inflammatory colon at high concentrations (So et al., 2007). To exam the drug release profile of PAE-S100 conjugate, the conjugate was dissolved in different pH environments which contained specific enzymes to mimic the real physiological conditions in gastric juice, small intestine and colon (see Figure 2). In addition, scanning electronic microscopic was used to observe the morphology of PAE-S100 in different physiological conditions to test the stability of the PAE-S100 microparticle. Finally, an animal study was performed to demonstrate the PAE-S100 efficacy in vivo. Mice were treated with dinitrochlorobenzene and acetic acid to induce UC. Administrated different formulations, PAE only, PAE-S100 and PAE enema, the mice were observed for changes in body weight, fecal condition, as well as pathological changes in colonic tissues. Myeloperoxidase (MPO) activity in the tissues was measured by ELISA kits to indicate tissue recovery in mice. All the information was used to evaluate the efficacy of PAE-S100 comparing to other formulations.

## 2. Materials and methods

### 2.1. Materials

Inosine, aminopropionic acid, bovine serum albumin, and hypoxanthine were supplied from Shanghai McLean Biochemical Technology Co. Ltd (Shanghai, China). Soybean lecithin was purchased from Beijing Biotechnology Co. Ltd (Beijng, China). Glyceryl monooleate was obtained from Shanghai Jiafashi Trading Co. Ltd (Shanghai, China). Methanol, ethanol, phenol reagent, ethyl ether, glacial acetic acid, NaC_4_H_4_O_6_, CuSO_4_, Na_2_CO_3_, NaOH were bought from Tianjin Fuyu Fine Chemicals Co. Ltd (Tianjin, China). Tianjin Bodi Chemical Co. Ltd (Tianjin, China) supplied Tween 80, paraffin, acetic acid, ninhydrin, ascorbic acid. Hydrochloric acid, phosphate buffer solution, acetic acid, sodium sulfate, dinitrochlorobenzene, normal saline, and formalin were purchased from Shanghai Yuanye Biotechnology Co. Ltd. (Shanghai, China). Coomassie bright blue R250 and SDS-PAGE gel preparation kit were purchased from Biyuntian Biotechnology Co. Ltd (Shanghai, China). Protein standard molecular weight marker 26610 was obtained from Beijing Solaibao Technology Co. Ltd (Beijing, China). ELISA kits were acquired from Shanghai Fanke Biotechnology Co. Ltd (Shanghai, China). Deionized water was filtered by 0.22 μm PES filter (Haining Jinzheng Filter Material Technology Co. Ltd, China) to remove any contaminants.

### 2.2. Mice

Animal Experiment Center in Shenyang Pharmaceutical University supplied SD mice (male, 200 ± 20 g). All animals were kept in the housing system under 24 ± 1 °C, relative humidity (40-70%), and 12-12 hours cycles of light-dark with free water and fodder access. Before the experiment, the animals were deprived of food for 12 hours but with free access to water. The protocols were performed strictly according to guidelines issued by Animal Experiment Center in Shenyang Pharmaceutical University.

### 2.3. Preparation of Periplaneta americana extract

The dried *Periplaneta americana* body was blended to fine powder and was soaked in 90 v/v % ethanol solution for 12 hours (Fu et al., 2021; Hua-ShengYong-Ming et al., 2021). Then the mixture was incubated in a water bath at 60 °C (Gongyi Yuhua Instrument Co. Ltd, Henan, China) for 1 hour and filtered by 0.22 μm PES filter. The ethanol was removed by vacuum evaporation (Shanghai Yarong Biochemical Instrument Co. Ltd, Shanghai, China) at 60 °C to obtain concentrated solution, and then the solution was filtered through 0.22 μm PES filter to remove insoluble impurities.

The macroporous adsorption AB-8 resin (Shanghai Acmec Biochemical Co. Ltd, Shanghai, China) was used to purify the PAE. The PAE was loaded into the packed column and eluted by 0.22 μm filtered deionized (DI) water at 100 mL/hour for 2 hours at room temperature. 95 v/v % ethanol was used for desorption at 100 mL/hour for 1.5 hours and the elution was collected. Finally, the PAE mixture was freeze-dried in a freeze-dryer (Ningbo Scientz Biotechnology Co. Ltd, Zhejiang, China) at −100 Pa, −40 °C for 24 hours.

### 2.4. Characterization of polypeptide in PAE

The protein content in PAE was detected by high performance liquid chromatooraphy (Hitachi Scientific Instruments (Beijing) Co. LTD). The mobile phase was 0.1 M Na2SO4, 0.05 M Na2HPO4 and 0.05 M NaH2PO4. The flow rate was 0.8 ml/min at 25 °C. The chromatographic column was TSKgel G3000SWxl (Yamasaki & Kato, 1989).(Dongcao (Shanghai) Biotechnology Co., LTD). Each injection amount is 10 µL and the peak time of protein at about 10 minutes.

### 2.5. Preparation of eudragit S100 PAE microparticles

6 g/L Eudragit S100 was prepared by dissolving Eudragit S100 in ethanol solution. 1 mg/mL PAE solution was prepared through dissolving PAE lyophilized cake in ethanol. Then 10 mL 6 g/L Eudragit S100 polymer solution was mixed with 1 mL 1 mg/mL PAE ethanol solution by sonicating (Ningbo Xinzhi Biotechnology Co. Ltd, Zhejiang, China) for 30 minutes to form fine emulsion. The mixture was then mixed with 0.33 g/L lecithin and 30.3 g/L glycerol monooleate in a blender (Ningbo Scientz Biotechnology Co. Ltd, Zhejiang, China) for 2 minutes to form 110 mL emulsion (Thakral et al., 2010). The emulsion was stirred at room temperature with a magnetic stir bar (Gongyi Yuhua Instrument Co. Ltd, Henan, China) at 420 rpm for 8 hours until the organic solvent completely evaporated. Finally, the microparticles were freeze-dried in a freeze-dryer (Ningbo Xinzhi Biotechnology, Zhejiang, China) at −100 Pa and − 40 °C for 24 hours and stored at 4 °C (Li et al., 2021).

### 2.6. Encapsulation yield rate of PAE

Polypeptide concentration was used to determine the encapsulation efficiency of PAE. 100 mg PAE was added to preparate the microparticles according to method 2.2.2. Then the solution was centrifuged (Shanghai Anting Scientific Instrument Factory, Shanghai, China) at 3000 rpm for 15 minutes, and supernatant was collected. The supernatant was used to calculate the concentration of polypeptides using method 2.3.1. The encapsulation efficiency (EE) of polypeptide microparticles was calculated according to (Thakral et al., 2010),

(1) $$EE=\frac{C_{total}-C_{free}}{C_{total}}\times 100\%$$

where

C_total_ was the amount of total polypeptide;

*C_free_* was the amount of free polypeptide that was not captured.

### 2.7. In vitro drug release experiments

250 mL simulate gastric solution was prepared by dissolving 0.1 mol/L HCl, and 10 g/L of pepsin into 0.22 μm filtered DI water. 250 mL simulate small intestine solution was prepared by dissolving 6.8 g/L of KH_2_PO_4_ and 10 mg/L of trypsin into filtered DI water, and 0.1 mol/L NaOH was used to adjust the pH of the solution to 6.8, and 250 mL simulate colonic fluid prepared by dissolving 0.41 g/L of KH_2_PO_4_ and 5.59 g/L K_2_HPO_4_. 4 mg/mL PAE-S100 microparticles were placed in three different solutions. They were agitated in an incubator (Jiangsu Keji Instrument Co. Ltd, Jiangsu, China) at 37 °C and 140 rpm for 20 hours, and 1 mL solution was taken out at 0.3, 0.6, 1, 1.5, 2, 3, 4, 5, 6, 7, 8, 9, 10, 11, 12, 13, 14, 15, and 20 hours, respectively (Thakral et al., 2010; Wang et al., 2016; Yuan et al., 2019), which were centrifuged at 5000 rpm (Shanghai Anting Scientific Instrument Factory, Shanghai, China). The supernatant was used to quantify the concentration of polypeptide. The sediment was collected, and its surface morphology was observed by SEM (Wang et al., 2016) (Shimadzu Corporation, Kyoto, Japan).

### 2.8. Drug release kinetics

To investigate the PAE release kinetics from the microspheres, the in vitro drug release data were fitted into various mathematical models as follows: zero-order (equation 2), first-order (equation 3), Korsmeyer-Peppas (equation 4) (Malipeddi et al., 2016; Yuan et al., 2019),

(2) $$Q_{t}=K_{1}t$$

(3) $$Q_{t}=1-e^{-K_{2}t}$$

(4) $$Q_{t}=K_{3}t^{n}$$

where

Qt denoted the Cumulative release (%) of PAE at different time;

K_1_, K_2_, and K_3_ denoted the release-rate constants for zero-order, first-order, and Korsmeyer-Peppas;n was the release exponent.

### 2.9. Sodium dodecyl sulfate-polyacrylamide gel electrophoresis (SDS-PAGE) analysis

The equipment was filled with sealing glue (1 mL distilled water, 1 mL 30 w/v % Acrylamide-Bis, 1 mL 1 M Tris-HCl pH 6.8, 0.04 mL 10 w/v % ammonium persulfate, 0.004 mL N,N,N,N-tetramethylethylenediamine (TEMED)) , separating glue (2.7 mL distilled water, 3.3 mL 30 w/v % Acrylamide-Bis, 1 mL 1 M Tris-HCl pH 8.8, 0.1 mL 10 w/v % SDS, 0.1 mL 10 w/v % ammonium persulfate, 0.004 mL TEMED) and concentrating glue. (2.7 mL distilled water, 0.7 mL 30 w/v % Acrylamide-Bis, 0.5 mL 1 M Tris pH 8.8, 0.04 mL 10 w/v % SDS, 0.04 mL 10 w/v % ammonium persulfate, 0.004 mL TEMED). 4 mg/mL PAE microparticles were prepared in simulated gastric solution, simulated small intestine juice and simulated colonic juice, respectively. They were agitated in an incubator 37 °C and 140 rpm for 24 hours, and centrifugated at 12000 rpm for 15 minutes to obtain supernatant. 35 μL supernatant was mixed with 15 μL loading buffer (250 mM Tris-HCl, 10 w/v % SDS, 0.5 w/v % bromophenol blue, 50 w/v % glycerin, 5 w/v % β-mercaptoethanol) in 1.5 mL tube. Then the liquid was boiled for 5 minutes and centrifuged at 12000 rpm for 5 minutes. 30 μL protein samples and protein marker were loaded into the gel. When bromophenol blue line was closed to sealing glue, the gel was collected and stained by Coomash bright blue R-250 for 12 hours. Then the gel was de colorized by eluent, and the images of gel were recorded (Simpson, 2007; Brunelle & Green, 2014; Abedi et al., 2022).

### 2.10. Induction of ulcerative colitis in mice

40 mice were fed in cages for a week. Each mouse took the spinal column as the dividing line, and the skin with an area of 1 cm × 2 cm on the symmetrical part of its back was taken as the experimental area. The hair of mice was removed to expose a 1 cm × 2 cm area on its back. 2 w/v % dinitrochlorophenone solution was used to sensitize this area for 14 consecutive days. On the 15th day, the mice were fasting for 12 hours, and a silicone tube was slowly inserted into the anus for 7 ∼ 8 cm. 0.25 mL 1 w/v % dinitrochlorobenzene alcohol solution was injected into mice through the tube. The mice were gently lifted upside-down for 30 seconds to prevent the outflow of the liquid. On the 16th day, 0.7 mL 6 v/v % acetic acid solution was injected into the mice using the method described above to induce UC (Aleksandra et al., 2016; Wang et al., 2016; Rezayat et al., 2018).

### 2.11. In vivo experiment in mice

40 mice were randomly divided into five groups, model UC group, PAE treatment group, PAE-S100 treatment group, PAE enema treatment group, and control group. The control group was healthy mice. Model UC group was mice that had UC without administration. PAE-S100 treatment group was the UC mice that were treated with 0.26 g PEA microparticles which were equal to 10 mg PAE. The PAE treatment group was the UC mice that were oral administrated with 10 mg PAE. PAE enema treatment group was the UC mice that were fed with 10 mg PAE by enema. Mice in each group were given the drug every day for 14 days consecutively and were fed normally (Wang et al., 2016).

### 2.12. Disease activity index of mice

During the treatment, the disease activity index score was evaluated by changes in body weight, stool consistency, and blood in feces (Wang et al., 2016; Patole & Pandit, 2018). The specific scoring methods were shown in Table 1.

**Table 1.** Evaluation of disease activity index (DAI).

Score	Weight loss	Stool consistency	Bleeding
0	None	Normal	No bleeding
1	1–5 %	Nomal	Slight bleeding
2	6–10 %	Nomal/soft	Slight bleeding
3	11–20 %	Soft	Slight bleeding
4	More than 20 %	Watery	Gross bleeding

### 2.13. Histopathology of colon

After 14 days of continuous administration, all mice were killed by cervical dislocation, and the liver and colon were collected for further analysis. The colon tissue was stained by hematoxylin-eosin (HE), and its morphology was observed under the microscope Olympus BX 60 (Olympus, Japan) with eyepiece 40 and objective 10 (Wang et al., 2016).

### 2.14. Myeloperoxidase (MPO) activity

The activity of MPO in tissues was determined by MPO ELISA kits (Franck et al., 2009). 50 mg colon tissue was homogenized in saline. The mixture was placed in a 10 mL centrifuge tube and centrifuged at 4 °C and 10000 rpm for 10 minutes. 5 mL supernatant was collected for further analysis. 40 μL sample dilution solution and 10 μL test sample solution were added into the well on the HRP-coated plate respectively. Then 100 μL HRP-Conjugate reagent were added at the bottom of the HRP-plate, except blank well. The plate was incubated at 37 °C for 30 minutes sealed with plate cover. The solution in the well was removed and the well was washed by washing solution for 5 times. Then 50 μL solution A and solution B was added in each well, and mixture was incubated at 37 °C for 15 minutes. Finally, 50 μL termination solution was added to each well to terminate the reaction. Then, the absorbance of each well was measured at 450 nm and the concentration of MPO was calculated based on the standard calibration curve.

### 2.15. Statistical analysis

All experiments were carried out in triplicate. The experimental data were analyzed using SPSS version 22.0 and Origin 2021. One-way analysis of variance and Tukey's post-hoc test were used to assess the normally distributed data, and the results are reported as mean ± SD. Statistical significance was accepted at *p < 0.05.

## 3 Results

### 3.1. Results of in vitro release studies

**Figure 3.**
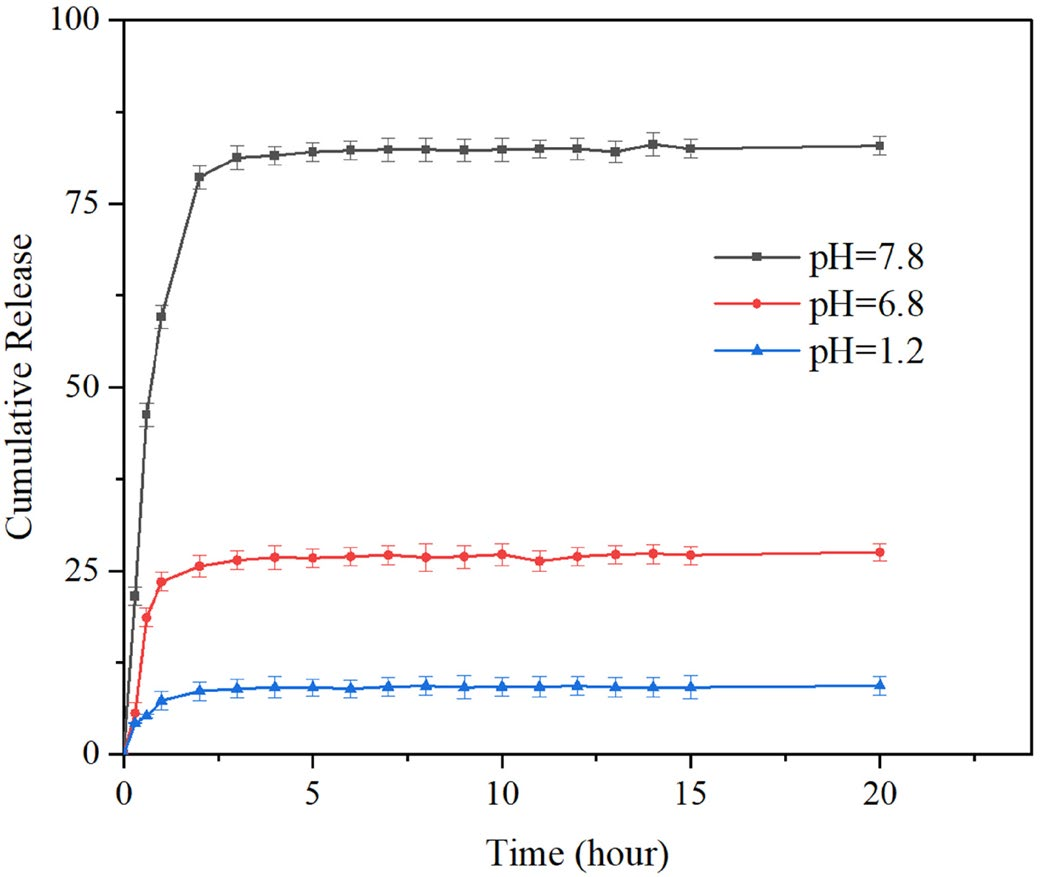
The percentage of drug released from PAE-S100 microparticles. PAE-S100 microparticles in the simulated gastric solution (pH 1.2) (■); PAE -S100 microparticles in the simulated small intestinal solution (pH 6.8) (●); PAE-S100 microparticles in the simulated colonic solution (pH 7.8) (▲).

**Figure 4.**
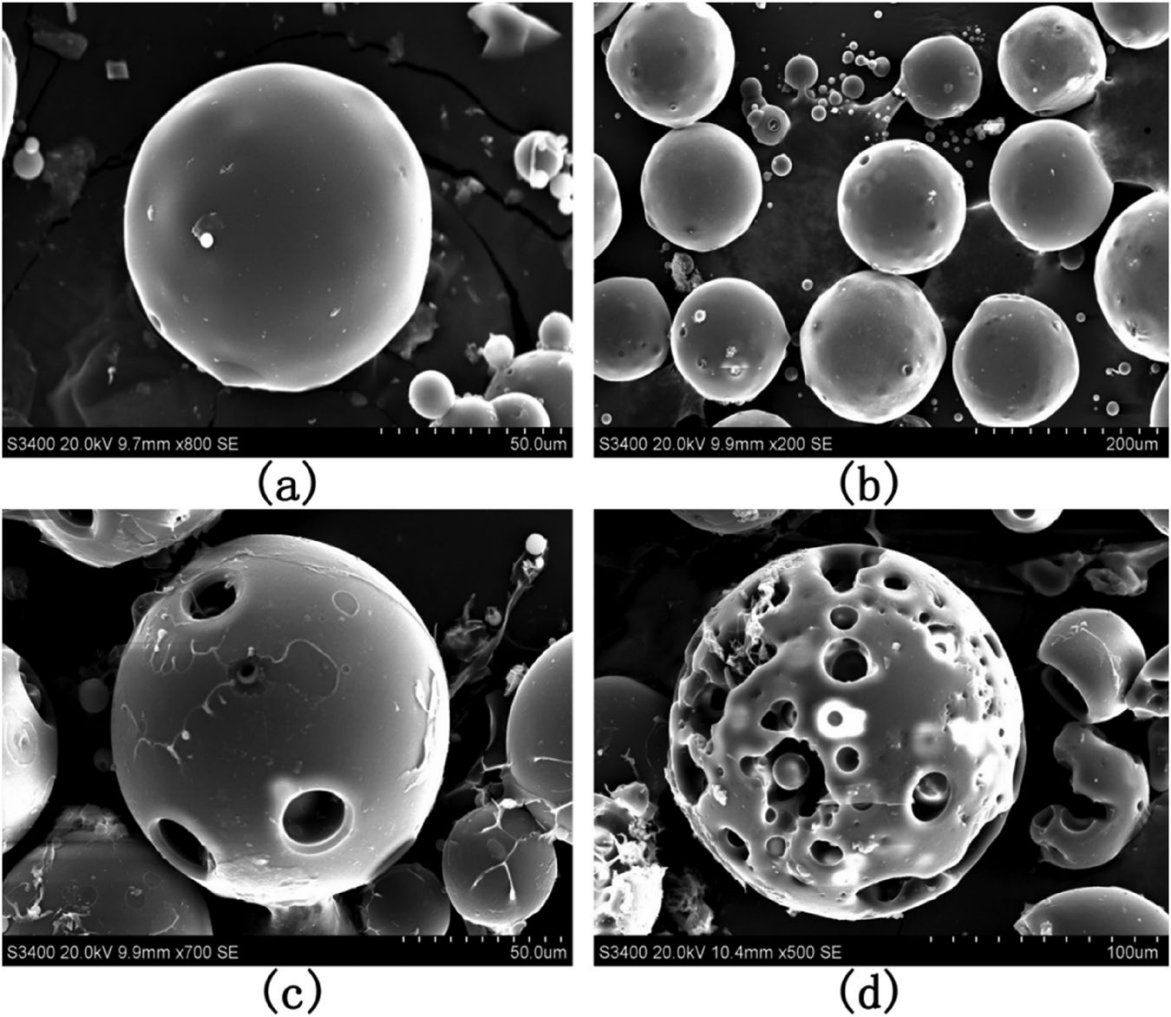
SEM images of PAE-S100 microparticles in different pH solutions. The morphology of (a) lyophilized microparticles; (b) the microparticles in simulated gastric solution at pH =1.2 for 18 minutes; (c) the microparticles in the simulate small intestine fluid at pH= 6.8 for 15 minutes;(d) the microparticles in simulate colonic fluid at pH= 7.8 for 2 hours.

To understand the drug release profile of PAE-S100 microparticles in the digestion system, the microparticles were placed in three different solutions which mimicked environments in stomach, small intestine and large intestine. Figure 3 showed that protein was initially released from microparticles in all conditions and the release rate reduced over time. The microparticles stopped releasing protein after 2 hours in all conditions. Only 9% total protein was released into the bulk solution at pH 1.2, which was much lower than the released amount at pH 6.8 (about 28%) and at pH 7.8 (85%). Figure 4a showed the PAE-S100 microparticles before dissolving into the solutions, and these microparticles were spherical and intact. After incubating at pH 1.2 for 2 hours, the majority of PAE-S100 microparticles were still intact and in a spherical shape (see Figure 4b), which was consistent with the result that only a small amount of protein was released. Figure 4c showed the morphology of PAE-S100 microparticles which were dissolved at pH 6.8, and only several cavities were observed on the surface of microparticles without major defect of the structure. In contrast, Figure 4d indicated that the structure of microparticles that were incubated at pH 7.8 started to collapse, which could cause the release of protein into bulk solution.

Three drug release kinetics models were used to study the release kinetics of microparticles. The results are shown in Table 2, which indicated that the release of PAE in microparticles was followed a first-order kinetics whose R^2^ is 0.98, 0.97, 0.99.

**Table 2.** Simulation regression of drug release curve.

Type	pH = 7.8	R^2^	pH = 6.8	R^2^	pH = 1.2	R^2^
Zero-order	y = 54.49 + 2.37x	0.31	y = 18.08 + 0.76x	0.28	y = 6.21 + 0.25x	0.32
First-order	y = 82.45(1-e^-1.44x^)	0.98	y = 27.02(1-e^-1.58x^)	0.97	y = 9.13(1-e^-1.62x^)	0.99
Korsmeyer-Peppas	y = 59.32×^0.14^	0.64	y = 19.72×^0.14^	0.57	y = 6.83×^0.13^	0.72

### 3.2. SDS-PAGE analysis

**Figure 5.**
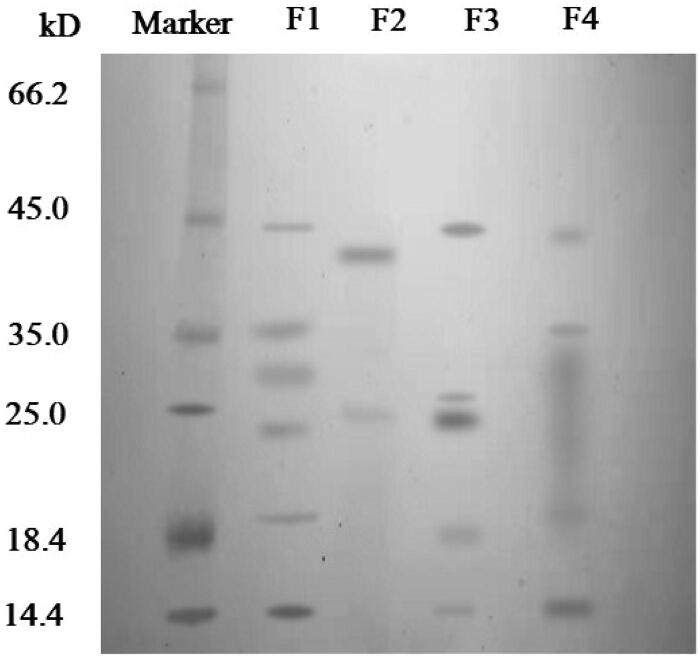
SDS-PAGE gel of marker, *Periplaneta americana* extract (F1), protein that was released form microparticles in simulated gastric solution (pH 1.2) for 2 hours (F2), protein that was released from microparticles in simulated small intestinal fluid (pH 6.8) for 2 hours (F3), protein in supernatant that was released from microparticles in simulated colonic fluid (pH 7.8) for 2 hours (F4).

Figure 5 showed the major protein species in different conditions. The electrophoretic band of a protein standard sample was shown in the first column. F1 was the electrophoretic band of protein in *Periplaneta americana* extract dissolved in buffer, which was mainly concentrated in 14.4 kDa, 18.4 kDa, 25 kDa, 25 kDa ∼35 kDa, 35 kDa and 45 kDa. F2 was the result of protein in supernatant that was released form microparticles in simulated gastric fluid (pH 1.2) for 2 hours. Only two clear bands were observed at about 25 kDa and 35 ∼ 45 kDa. Since the microparticles were still intact (see Figure 4b) and the majority of the protein was still encapsuled in the microparticle. Only some protein that was attached on the surface might release in the solution. In addition, compared to F1, a new band at 35 ∼ 45 kDa was observed, because pepsin was added into the solution to mimic the gastric fluid, whose molecular weight was 35 ∼ 45 kDa. F3 was the electrophoretic band of protein in supernatant that was released from microparticles in simulated small intestinal fluid (pH 6.8) for 2 hours. In this case, only part of microparticles have been decomposed (see Figure 4c) and small amount of protein was leaked into the small intestinal fluid. So F3 band only showed 4 protein species that were similar to F1. Furthermore, we did see a new type of protein in F3, but it might be trypsin (about 25 kDa) that was used to simulate small intestine fluid. F4 was the electrophoretic band of protein in supernatant that was released from microparticles in simulated colonic fluid (pH 7.8) for 2 hours. The structure of microparticles was decomposed completely (see Figure 4d), and most of components were dissolved in the solution. A wide band from 18.4 to 35 kDa was found, which indicated that some protein species were broken down by trypsin at pH 7.8.

### 3.3. Disease activity index (DAI) of mice

**Figure 6.**
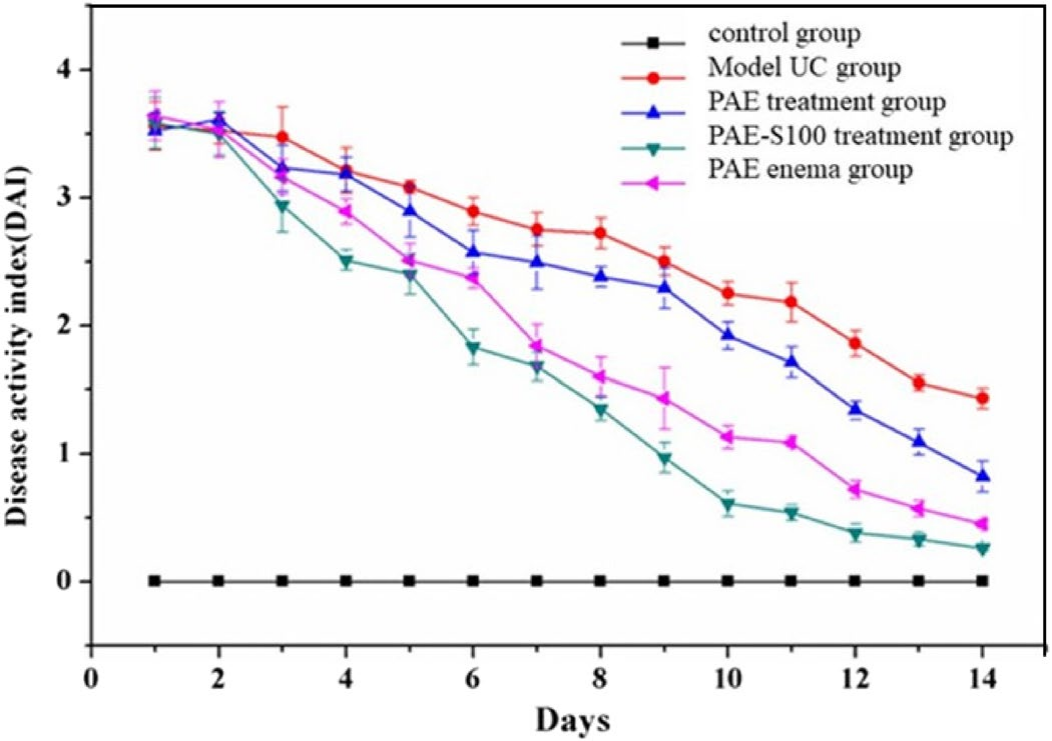
Changes in disease activity index score of mice in 14 Days. The activity index of control group (■), model UC group (●), PAE only treatment group (▲), PAE-S100 treatment group (▼), PAE enema group (◄).

Disease activity index was visual data that could reflect the recovery of mice. All groups had high DAI scores due to UC at day 0 except the control group (see Figure 6), and DAI scores started to decline over time which indicated that mice could recover from UC. After 14 days, the model UC group had the highest DAI score and the mice still had symptoms of UC. The PAE treatment group had lower DAI score than model UC group, but the score was still higher than the other groups. The mice still suffered with UC, but they were slowly recovered over time. Because most active components in PAE was degraded during the digestion process, and only a small amount of PAE could reach colon, which significantly reduced the drug efficacy. When the PAE was delivered through enema without passing through stomach and small intestine, the mice were cure faster than oral administrated method and symptoms were gradually relieved. Even though the drug could target the colon directly using enema, the patients might suffer a lot during the operation, which limited the use of this method. The mice which were oral administrated PAE-S100 were recovered fastest among all test groups, and all symptoms were significantly reduced. This result was consistent with the observation of in vitro experiment that PAE-S100 could only release formulation at colon.

### 3.4. Myeloperoxidase (MPO) activity

MPO activity could be used to predict the level of inflammatory in colon. As shown in Table 3, the activity of MPO in the control group was 0.22 ± 0.06 U/g, which indicated this group of mice was healthy and had no inflammation. The model UC group had the highest MPO activity, 0.87 ± 0.153 U/g, since the colon was still injured and seriously inflamed after 14 days without any treatment. The oral administrated PAE group had similar MPO activity level to model UC group, but higher than the other two groups. Small portion of PAE might reach the colon and reduced the MPO activity, but the drug concentration was too low to treat the disease. When the PAE was delivered to colon though enema or PAE-S100 system, the MPO activity level in the colon was significant reduced. This proved that oral administrate PAE-S100 formulation could bypass the harsh digestion process and reach colon to deliver the drug.

**Table 3.** MPO activity in mice.

Group	MPO (U/g)
Blank control	0.22 ± 0.060
Model UC control	0.87 ± 0.15
PAE enema treatment	0.37 ± 0.10
PAE treatment	0.68 ± 0.14
PAE-S100 treatment	0.32 ± 0.090

### 3.5. Histopathological observation of colon

**Figure 7.**
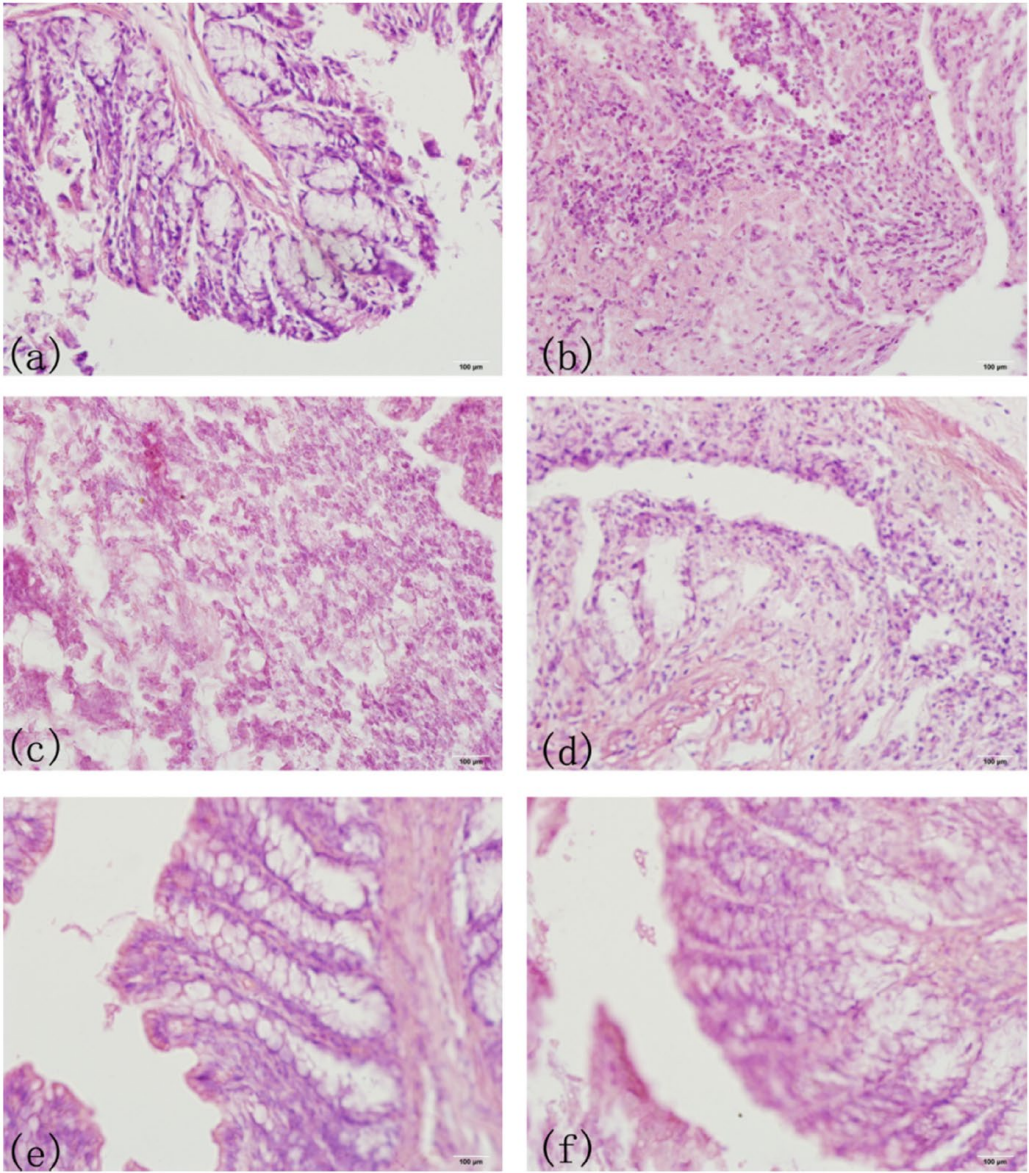
HE Staining of Mice Colon Sections. The complete villus structure and normal goblet cell morphology in normal colon of mice(a); colon biopsy of model UC group in the first day; the colon lesions of model UC group in the 14th day (c); colon of PAE treatment group in the 14th day (d); colon of PAE enema group in the 14th day; colon of PAE-S100 treatment group in the 14th day (f).

As shown in Figure 7a exhibited the complete villus structure and normal goblet cell morphology in normal functional colon of mice. Figure 7b showed colon biopsy of model UC group in the first day, and the colon lost its villus structure and epithelial cells in the tissue. Figure 7c showed the colon lesions of model UC group in the 14th day, and there were still many inflammatory cells, which reflected the colon injury had not been recovered. Figures 7d, 5e and f showed the PAE treatment group, PAE enema group and PAE-S100 treatment group in the 14th day, the villi had regenerated and the number of inflammatory cells had decreased. However, Figure 7e and f showed that the colon did recover from the UC when the drug was directly delivered to colon.

## 4. Discussion

Wound healing is a complex biological process involving hemostasis, inflammatory control, proliferation, and tissue remodeling (Kasuya & Tokura, 2014). When tissue damage occurs, the coagulation pathway is triggered, leading to the formation of a temporary fibrin matrix that allows cells to migrate to the site of injury. At the same time, platelet-derived factors attract white blood cells, activating inflammatory responses. Platelets and immune cells then secrete growth factors and cytokines that promote wound reepithelialization, extracellular matrix deposition, and angiogenesis. Active components in PAE promotes the proliferation and migration of human keratin cells, HaCaT cells (Song et al., 2017), and the expression of epidermal growth factor and vascular endothelial growth factor (Lekic & McCulloch, 1996; Velazquez, 2007; Li et al., 2019), which accelerate the rate of healing.

The oral administrated PAE has to pass through the gastrointestinal tract, which is composed of hydrochloric acid, protein-digesting enzymes and mucus, to access the inflammatory site in colon (Brown et al., 2020). However, under such highly harsh conditions, protein, one of the active components in PAE, is easily broken down into small polypeptide pieces and amino acids and lose its function (Hamman et al., 2005; Brown et al., 2020). Therefore, only a small fraction of the components can reach the colon which limited the efficacy of PAE to treat UC. Eudragit S100 (see Figure 1) is an anionic polymer formed by copolymerization of methacrylic acid with methyl methacrylate (1:2). When the microparticle which contains both Eudragit S100 and PAE arrive in the stomach, the Eudragit S100 can prevent acid and enzyme from penetrating into the core and degrading the PAE. Reaching in the colon, where the pH ranges from 5 to 8, carboxyl groups of Eudragit S100 react with alkali or amines and form water-soluble salts (Thakral et al., 2013). Eudragit S100 start to decompose gradually due to the pH, and PAE was released in colon. Therefore, Eudragit S100 can be used as a drug carrier to create a pH-dependent colon-targeted oral drug delivery system (Khan et al., 2000; Subudhi et al., 2015), which can prolong the residence time of the drug in the gastrointestinal tract and improve oral PAE absorption (Al-Kassas et al., 2021).

Myeloperoxidase (MPO), one of the most important members of the peroxidase family, mainly exists in neutrophils and mononuclear macrophages (Podrez et al., 2002). When inflammation occurs in the body, MPO in neutrophils is released into the circulatory system and participates in the inflammatory response of the body. Therefore, the level of MPO can be used to evaluate the degree of intestinal injury (Ogawa et al., 2002; Kiyosue et al., 2006; Li et al., 2008; Kim et al., 2016; de Oliveira et al., 2021). The high concentration of MPO reflects that the tissue is inflamed. When PAE-S100 microparticles arrived at colon, Eudragit S100 started to decomposed and PAE were released in the environment. Studies had shown that PAE had antimicrobial activity against bacteria (Kim et al., 2016) by inducing oxidative stress to activate apoptotic signals in mitochondria and promote antifungal activity (Yun et al., 2017). The PAE could inhibit TGF-β1, NF-κB,α-SM, and TIMP-1, thereby blocking relevant signaling pathways and preventing inflammatory responses to attenuate (Dingchun et al., 2018). PAE could also decrease the expression of IL-1β, IL-6, IFN-γ, and TNF-α which inhibited the PI3K/AKT/NF-κB signaling pathway, decreasing the expression of inflammatory cytokines (Ni et al., 2022). However, it is hard to determine which active component in PAE play a dominant role in curing UC due to abundant compositions in the PAE. In the future study, all the active components in PAE will be separated and collected to test their effectiveness in treatment of UC and investigate the potential of practical use in clinic.

## 5. Conclusions

In this study, we used Eudragit S100 as a drug carrier to deliver PAE in colon. PAE-S100 microparticles could research the colon without degradation in the digestion process and release PAE in colon to treat UC. Both DAI score and MPO were significantly reduced using the PAE-S100 drug delivery system. In addition, this system could be potentially used for other oral protein formulation administration.

## Author contributions

All authors contributed to this paper, with Meng Li taking the supervision and writing the original draft; Hao Wu reviewing and checking the content and editing the final version of the manuscript; Jing Han, Yang Han designing the framework and research, conceptualization;Hao Wu, Meng Li, Shuang Wang, Shengshun Wu conducting the experimental operation; All authors contributed extensively to this work. All authors have read and agreed to the published version of the manuscript.

## Supplementary Material

Supplemental Material

Supplemental Material
