# Supplementary figures and images for: Development of microparticles for oral administration of Periplaneta americana extract to treat ulcerative colitis
Source: Drug Deliv. 2022 Aug 18;29(1):2723–33. doi: 10.1080/10717544.2022.2112115 (PMC9521608; doi:10.1080/10717544.2022.2112115)

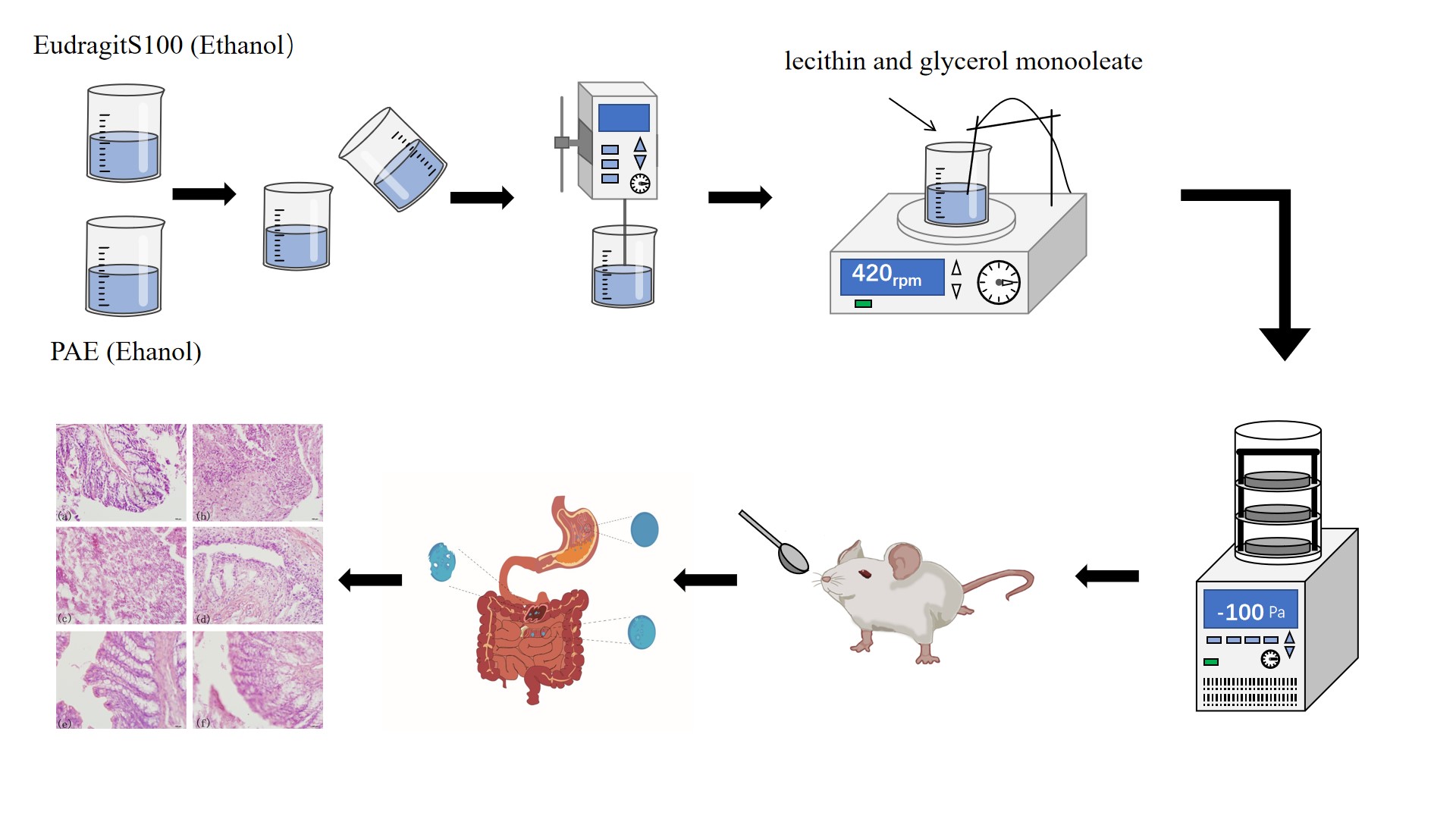

Supplement: Supplemental Material [file IDRD_A_2112115_SM3254.jpg]
