# Supplementary material for: Development of microparticles for oral administration of Periplaneta americana extract to treat ulcerative colitis
Source: Drug Deliv. 2022 Aug 18;29(1):2723–33. doi: 10.1080/10717544.2022.2112115 (PMC9521608; doi:10.1080/10717544.2022.2112115)
Supplement: Supplemental Material [file IDRD_A_2112115_SM3253.docx]

Eudragit S100 polymer solution was mixed with PAE ethanol solution by sonicating. Then mixture was then mixed with lecithin and glycerol monooleate in a blender . The emulsion was stirred at room temperature with a magnetic stir bar at 420 rpm. Them the microparticles were freeze-dried in a freeze-dryer. Finally, When mice were treated with this drugs. the drugs were not decomposed in stomach and small intestine but damaged in the colon, which had a good curative effeet on colitis.
